# Late Neogene and Early Quaternary Paleoenvironmental and Paleoclimatic Conditions in Southwestern Europe: Isotopic Analyses on Mammalian Taxa

**DOI:** 10.1371/journal.pone.0063739

**Published:** 2013-05-23

**Authors:** Laura Domingo, Paul L. Koch, Manuel Hernández Fernández, David L. Fox, M. Soledad Domingo, María Teresa Alberdi

**Affiliations:** 1 Earth and Planetary Sciences Department. University of California Santa Cruz, Santa Cruz, California, United States of America; 2 Departamento de Paleontología, Universidad Complutense de Madrid, Madrid, Spain; 3 Departamento de Cambio Medioambiental, Instituto de Geociencias (UCM, CSIC), Madrid, Spain; 4 Department of Earth Sciences. University of Minnesota, Minneapolis, Minnesota, United States of America; 5 Museum of Paleontology, University of Michigan, Ann Arbor, Michigan, United States of America; 6 Departamento de Paleobiología, Museo Nacional de Ciencias Naturales-CSIC, Madrid, Spain; Ludwig-Maximilians-Universität München, Germany

## Abstract

Climatic and environmental shifts have had profound impacts on faunal and floral assemblages globally since the end of the Miocene. We explore the regional expression of these fluctuations in southwestern Europe by constructing long-term records (from ∼11.1 to 0.8 Ma, late Miocene–middle Pleistocene) of carbon and oxygen isotope variations in tooth enamel of different large herbivorous mammals from Spain. Isotopic differences among taxa illuminate differences in ecological niches. The δ^13^C values (relative to VPDB, mean −10.3±1.1‰; range −13.0 to −7.4‰) are consistent with consumption of C_3_ vegetation; C_4_ plants did not contribute significantly to the diets of the selected taxa. When averaged by time interval to examine secular trends, δ^13^C values increase at ∼9.5 Ma (MN9–MN10), probably related to the Middle Vallesian Crisis when there was a replacement of vegetation adapted to more humid conditions by vegetation adapted to drier and more seasonal conditions, and resulting in the disappearance of forested mammalian fauna. The mean δ^13^C value drops significantly at ∼4.2−3.7 Ma (MN14–MN15) during the Pliocene Warm Period, which brought more humid conditions to Europe, and returns to higher δ^13^C values from ∼2.6 Ma onwards (MN16), most likely reflecting more arid conditions as a consequence of the onset of the Northern Hemisphere glaciation. The most notable feature in oxygen isotope records (and mean annual temperature reconstructed from these records) is a gradual drop between MN13 and the middle Pleistocene (∼6.3−0.8 Ma) most likely due to cooling associated with Northern Hemisphere glaciation.

## Introduction

Profound paleoenvironmental and paleoclimatic events in the late Cenozoic affected life on Earth and gave rise to modern climate regimes and biomes. Progressive cooling, which began in the middle Miocene (14-13.8 Ma), ultimately led to the onset of Northern Hemisphere glaciation ∼2.7 Ma [1]–[3]. This cooling was not monotonic, however. For example, reorganized ocean circulation, perhaps associated with initial restriction of circulation between the Pacific and Atlantic, contributed to the Pliocene Warm Period between ∼4.7 and 3.1 Ma [4]. Shifts in temperature and ocean circulation were associated with shifts in the global water budget, though impacts varied by region. Furthermore, terrestrial environments were transformed from the end of the Miocene to the beginning of the Pliocene (∼8-3 Ma) by the worldwide expansion of C_4_ plants [5]–[6]. C_4_ plants evolved repeatedly from C_3_ plants, most likely as a response to low atmospheric pCO_2_, higher temperatures and increasing water-stress [7].

In southern Europe, our focus here, tectonic closure of the Mediterranean Basin reduced circulation from the Atlantic, likely exascerbated by a drop in sea level associated with increased Antarctic ice volume, culminating with the formation of thick evaporite deposits (Messinian Salinity Crisis or MSC) between ∼6.0 and 5.3 Ma [8]–[9].

As one of the few locations in southern Europe with a relatively complete (albeit low resolution) late Cenozoic stratigraphic succession, a number of recent investigations have reconstructed regional paleoclimatic and paleoenvironmental conditions on the Iberian Peninsula. Based on the bioclimatic analysis of Plio-Pleistocene fossil rodent assemblages, Hernández Fernández *et al.* [10] argued there was a cooling trend, from subtropical temperatures in the early Pliocene to temperate conditions for the rest of the studied period. Study of palynological records from different Iberian sections led Jiménez-Moreno *et al.* [11] to suggest that warm temperatures of the Early to Middle Miocene gave way to progressively cooler temperatures in the remainder of Miocene and Pliocene. By the end of the Pliocene and beginning of the Pleistocene, the Iberian palynological record showed the development of steppes, coincident with cooler and drier conditions at the start of glacial-interglacial cycles in the Northern Hemisphere. Van Dam [12] investigated precipitation rates in the Iberian Peninsula using micro-mammal community structure. The most striking features are a decrease of mean annual precipitation (MAP) in the beginning of the Late Miocene (∼11−8.5 Ma), an increase in MAP in the middle part of the Late Miocene (∼8.5−6.5 Ma) and a drop in MAP between the end of the Late Miocene and the Late Pliocene (∼6.5−3 Ma). Böhme *et al.* [13] reconstructed MAP using herpetological assemblages between the end of the Early Miocene and the Early Pliocene in the Calatayud-Daroca Basin. Their MAP record differed from that of van Dam [12], with an increase in MAP at the beginning of the Late Miocene (∼11−9.7 Ma), a sharp decrease at ∼ 9.7 Ma, a progressive increase in MAP up to the middle Late Miocene (∼8.3 Ma) and a gradual decrease until the beginning of the Pliocene (∼5.4 Ma).

Mammalian tooth enamel is a reliable source of isotopic data that can be used to explore past environmental and climatic changes. Here, the stable carbon and oxygen isotope compositions of fossil tooth enamel from different genera of herbivorous mammals spanning from late Miocene to middle Pleistocene (∼11.1-0.8 Ma) were analyzed. Our objectives are twofold: 1) to infer the paleoecology of the selected taxa over the study interval, and 2) to reconstruct paleoenvironmental and paleoclimatic trends in Iberia from the late Miocene to the middle Pleistocene.

## Materials and Methods

**Figure 1:**
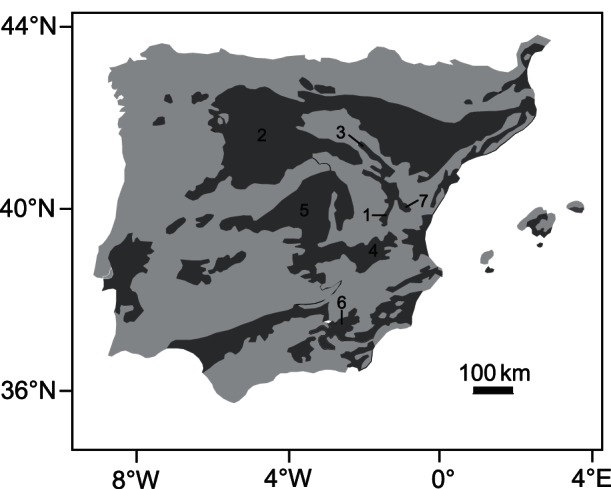
Situation of the studied fossil sites. Cenozoic basins of the Iberian Peninsula (dark grey) and situation of the basins where the fossil sites are located. 1–Teruel Basin, 2–Duero Basin, 3–Calatayud-Daroca Basin, 4–Cabriel Basin, 5–Tajo Basin, 6–Guadix-Baza Basin, 7–Sarrión-Mijares Basin.

The Iberian Cenozoic basins (Fig. 1) were formed as a consequence of Alpine compression between the African and Eurasian tectonic plates [14]–[15]. Most of the basins are located on basement comprising Precambrian and Paleozoic metasediments or granitoids and Mesozoic detrital and carbonate rocks. These basins constitute 40% of the total surface area of the Iberian Peninsula and they offer a complete sedimentary record that spans most of the Cenozoic. Most fossil sites selected for this study (La Roma 2, Masía de la Roma 604B, Puente Minero, Los Mansuetos, Cerro de la Garita, El Arquillo 1, Las Casiones, Milagros, La Gloria 4) are in the Teruel Basin in the northeastern Iberian Peninsula. The name, age and taxonomic composition for localities in the Teruel Basin and other Neogene and Quaternary sites are supplied in Table 1.

**Table 1:** Site, basin, MN, age (Ma) and taxa from this study.

Site	Basin	MN	Age (Ma)	*Equus stenonis*		*Mammuthus meridionalis*	*Elephas antiquus*	*Anancus arvernensis*	*Zygolophodon turicensis*	*Tetralophodon longirostris*	*Gomphotherium angustidens*	Undetermined Gomphotheriidae	*Gazella borbonica*	aff. *Gazella* sp. nov.	*Gallogoral meneghini*	*Gazellospira torticornis*	cf. *Hesperidoceras merlae*	*Protoryx* sp.	*Tragoportax amalthea*	*Tragoportax ventiensis*	*Tragoportax gaudryi*	*Tragoportax* sp.	*Hispanodorcas torrubiae*	Undetermined Bovidae	*Croizetoceros ramosus*	*Eucladoceros senezensis*	*Croizetoceros pyrenaicus*	*Pliocervus turolensis*	*Turiacemas concudensis*	*Palaeoplatyceros hispanicus*	Undetermined Cervidae	*Birgerbohlinia schaubi*	*Microstonyx major*
Huéscar 1	Guadix-Baza	MP	0.80		2	1	2																										
La Puebla de Valverde	Sarrión-Mijares	MN17	2.13										1		1									1	3								
Huélago	Guadix-Baza	MN16	2.60													3	2							4		2					8		
Huéscar 3	Guadix-Baza	MN15	3.70					2																									
Layna	Tajo	MN15	3.91										6																				
La Gloria 4	Teruel	MN14	4.19											4				4															
Venta del Moro	Cabriel	MN13	5.69																3	8							3						
Milagros	Teruel	MN13	5.69																			9											
Las Casiones	Teruel	MN13	6.08																			10											
El Arquillo 1	Teruel	MN13	6.32																			1		3				10					
Cerro de la Garita	Teruel	MN12	7.01						2	4											7								2				1
Los Mansuetos	Teruel	MN12	7.01																				4										
Puente Minero	Teruel	MN11	7.83							3											10											3	1
Masía de la Roma 604B	Teruel	MN10	8.26									2																					
La Roma 2	Teruel	MN10	8.79																		7												
Los Valles de Fuentidueña	Duero	MN9	9.55							15																							
Nombrevilla 1	Calatayud-Daroca	MN9	10.87							5																							
Cerro del Otero	Duero	MN7/8	11.13								3																			6			

Numbers indicate the presence and number of specimens analyzed in each locality. MP is middle Pleistocene. Age from Domingo *et al.* ([16], unpublished data).

The stable carbon and oxygen isotope composition of tooth enamel was analyzed for proboscideans, suids, giraffids, cervids, bovids, and equids from 18 localities from the Iberian Peninsula spanning from 11.1 to 0.8 Ma (late Miocene-middle Pleistocene) (Table S1). Chronological ages of the studied localities are from Domingo et al. ([16] and unpublished data). Although ages are assigned for each fossil site, the MN (Mammal Neogene) biochronology is used in order to allow comparisons among localities [17]–[21]. Since all the basins studied here belong to the same biogeographic province [22], the use of the MN units to aggregate fossil sites is assumed to be an appropiate approach, despite the fact that the Mammal Neogene biochronological system has been challenged as a true biozonation at larger scales [22]–[24].

Tooth enamel was sampled using a rotary drill with a diamond-tipped dental burr. Fossil teeth for this study are housed in the Museo Nacional de Ciencias Naturales-CSIC (Madrid, Spain) and Fundación Conjunto Paleontológico de Teruel-Dinópolis (Teruel, Spain), after being recovered in excavations carried out with public funding. Sampling was performed with the permission of both institutions.

Measurement of δ^13^C values of fossil tooth enamel allows for characterization of the diet of extinct taxa, providing a means to reconstruct past landscapes and habitats [25]–[31]. For herbivorous mammals, the δ^13^C value of tooth enamel (δ^13^C_enamel_) has a direct relationship to the δ^13^C value of the diet (δ^13^C_diet_), which varies depending on plant photosynthetic pathways (C_3_, C_4_, CAM), as well as ecological factors (aridity, canopy density, etc.) that affect fractionation during photosynthesis [32]–[33]. The δ^18^O values in the carbonate and phosphate fractions of mammalian tooth enamel record the δ^18^O value of body water (δ^18^O_bw_), which in turn is a reflection of oxygen uptake (inspired O_2_ and water vapor, drinking water, dietary water, oxygen in food dry matter) and loss (excreted water and solids, expired CO_2_, and water vapor) during tooth development [34]–[35]. Carbon and oxygen isotope results are reported in δ-notation δ^H^X_sample_ = [(R_sample_–R_standard_)/R_standard_]×1000, where X is the element, H is the mass of the rare, heavy isotope, and R = ^13^C/^12^C or^ 18^O/^16^O. Vienna Pee Dee Belemnite (VPDB) is the standard for δ^13^C values, and δ^18^O values are reported relative to Vienna Standard Mean Ocean Water (VSMOW).

Tooth enamel samples (n = 149) were analyzed for the carbon and oxygen isotope composition of carbonate in bioapatite (δ^13^C and δ^18^O_CO3_, respectively). Carbonate analyses were conducted at the stable isotope laboratories of the University of California Santa Cruz using a ThermoScientific MAT253 dual inlet isotope ratio mass spectrometer coupled to a ThermoScientific Kiel IV carbonate device and of the University of Minnesota using a ThermoScientific MAT252 dual inlet isotope ratio mass spectrometer coupled to a ThermoScientific Kiel II carbonate device. Approximately 5–6 mg of tooth enamel were sampled and treated with 30% H_2_O_2_ for 24 h. Samples were rinsed 5 times in deionized (DI) water and soaked for 24 h in 1 M acetic acid buffered to ∼pH 5 using Ca acetate solution. After 5 rinses with DI water, the resulting solid was freeze-dried at −40°C and at a pressure of 25×10^−3^ Mbar for 24 h. The standards used were Elephant Enamel Standard (EES, δ^13^C = −7.8‰ and δ^18^O = 1.6‰), Carrara Marble (CM, δ^13^C = 1.97‰ and δ^18^O = −1.61‰), NBS−18 (δ^13^C = −5.03‰ and δ^18^O = −23.01‰) and NBS-19 (δ^13^C = 1.95‰ and δ^18^O = −2.20‰). The standard deviations for repeated measurements of EES (n = 5), CM (n = 18), NBS-18 (n = 11) and NBS-19 (n = 6) were 0.06‰, 0.03‰, 0.04‰ and 0.08‰ for δ^13^C, respectively, and 0.19‰, 0.10‰, 0.05‰ and 0.08‰ for δ^18^O, respectively. Duplicate analyses were carried out for ∼10% of the samples (n = 15). The average absolute differences for δ^13^C and δ^18^O_CO3_ values were 0.04‰ and 0.38‰, respectively, and the standard deviations of these average differences were 0.15‰ and 0.29‰ for δ^13^C and δ^18^O_CO3_ values, respectively.

The δ^18^O values of phosphate in bioapatite (δ^18^O_PO4_) were measured on 149 enamel samples. Analyses were performed at the stable isotope laboratories of the University of California Santa Cruz using a ThermoFinnigan Delta plus XP IRMS coupled to a ThermoFinnigan High Temperature Conversion Elemental Analyzer (TCEA) and of the University of Kansas using a Thermo Finnigan MAT 253 IRMS coupled to a ThermoFinnigan TCEA. The chemical treatment is described in ÓNeil *et al.* [36] and Bassett *et al.* [37]. Between 1.5 and 2 mg of tooth enamel were recovered and dissolved in 100 µl of 0.5 M HNO_3_. 75 µl of 0.5 M KOH and 200 µl of 0.36 M KF were added to neutralize the solution and to precipitate CaF_2_ and other fluorides, respectively. Samples were then centrifuged and after removing the resulting solid, 250 µl of silver amine solution (0.2 M AgNO_3_, 0.35 M NH_4_NO_3_, 0.74 M NH_4_OH) was added and the samples were maintained at 50°C overnight to precipitate Ag_3_PO_4_. The resulting Ag_3_PO_4_ crystals were recovered by centrifugation and rinsing with DI water (5 times), after which vials were placed in an oven overnight at 50°C. The standards used were Fisher standard (δ^18^O = 8.4‰), Ellen Gray-UCSC High standard (δ^18^O = 19.0‰), Kodak standard (δ^18^O = 18.1‰) and NIST 120c (δ^18^O = 21.8‰). The standard deviations for repeated measurements of Fisher Standard (n = 48), Ellen Gray-UCSC High standard (n = 16), Kodak standard (n = 11) and NIST 120c (n = 15) were 0.5‰, 0.4‰, 0.7‰ and 0.4‰, respectively. Duplicate δ^18^O_PO4_ analyses were carried out on ∼ 30% of the samples. The average absolute difference for δ^18^O_PO4_ was 0.09‰ and the standard deviation of this average difference was 0.23‰.

To construct δ^13^C, δ^18^O_CO3_ and δ^18^O_PO4_ temporal trends, we have grouped our localities by MN and we calculated the weighted mean of isotopic values according to the following equation:
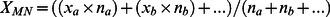
(1)where X_MN_ is the mean isotopic value (δ^13^C, δ^18^O_CO3_, δ^18^O_PO4_) for each MN, x_a_ and x_b_ are mean isotopic values for taxa a and b, and n_a_ and n_b_ are the number of selected teeth for taxa a and b. We opted to use the weighted mean since the number of analyzed teeth differs among taxa and therefore, they do not contribute equally to the final average. The application of the weighted mean when constructing temporal trends allows to avoid biases due to differences in physiological and ecological traits among taxa.

MAP was estimated following the work of Kohn [38] after a modern equivalent of diet composition (δ^13^C_diet, meq_) had been calculated using the following equation:
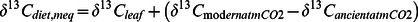
(2)where δ^13^C_leaf = _δ^13^C_tooth_ –14.1‰ [39], δ^13^C_modern atmCO2_ is −8‰, and δ^13^C_ancient atmCO2_ is the mean δ^13^C_atmCO2_ values from Tipple *et al.* [40] considering the following time bins: late Miocene, Pliocene and Pleistocene (Table S2).

The δ^18^O value of the water (δ^18^O_w_) ingested by fossil mammals was calculated using fossil mammal tooth enamel δ^18^O_PO4_ values and equations established for modern mammals (Table S3). Equations were selected according to the closest living relative of the fossil taxa assuming there were no significant differences in the δ^18^O_PO4_-δ^18^O_w_ fractionation between modern and fossil mammals.

Finally, we used a regression equation between MAT and weighted δ^18^O_w_ estimated using meteorological data included in Rozanski *et al.* [41]:



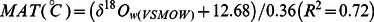
(3)


Equation 3 was selected because it uses data from meteorological stations worldwide, hence all existing climate regimes are represented. Tectonic reorganization including the closure and opening of sea gateways (e.g., closure of the Panama Isthmus and the passage between the Indian Ocean and the Tethys, opening of the Drake passage and Bering Strait), the uplift of mountain chains (e.g., Himalaya, Andes, Alps) along with shifts in the orbital cycles have exerted an important control on global ice volume and distribution as have perturbations in the atmospheric CO_2_ concentration and, by extension, in the carbon cycle. These factors have given rise to different climate regimes since the late Miocene and have culminated in modern climate configuration. In general, Cenozoic climates were globally warmer than at present as corroborated by different proxies [1], [42]–[44]. Warmer conditions have also been recorded in Western Europe during the Miocene and most of the Pliocene based on palynology, vertebrate fossils and General Circulation Models [11], [42], [45]–[46] with the definitive establishment of the Mediterranean climate regime at some point between 3.4 and 2.5 Ma [10]–[11]. Hernández Fernández *et al.* [10] and van Dam [12] highlighted the migration of the atmospheric cells, with the subtropical high pressure belt (between the Ferrel and Hadley cells) fluctuating since the late Miocene and profoundly affecting the distribution of Iberian ecosystems. Biome analyses carried out in the Iberian Peninsula between the Miocene and Pleistocene based on macro- and micro-mammals assemblages [10], [47]–[48] detected a shift in biomes from tropical deciduous woodland, savanna and subtropical desert during the Miocene and Early Pliocene, to nemoral broadleaf deciduous forest for the Late Pliocene, to the modern Mediterranean conditions characterized by schlerophyllous woodland-shrubland since the end of the Pliocene. Due to the different climate regimes and biomes that existed in the Iberian Peninsula during the period under study (late Miocene-middle Pleistocene), it is necessary to use a MAT-δ^18^O_w_ relationship that considers data from a wide range of climate regimes and biomes.

Statistical analyses were performed using SPSS PASW Statistics 18.0 software. Analysis of covariance (ANCOVA) was used to compare linear regressions. Analysis of variance (ANOVA) and Student-t tests were used to detect significant differences in isotopic data among taxa within MN intervals, whereas ANOVA and post-hoc Tukeýs analyses were used to analyze the variability of the isotopic record among MNs.

## Results and Discussion

### Diagenesis

The potential for diagenetic alteration should be assessed before accepting paleoecological or paleoenvironmental interpretations based on stable isotope results from fossil bioapatite. Here, only tooth enamel was analyzed, as it is the mineralized tissue least likely to experience isotopic alteration during diagenesis [49]. Phosphate oxygen is more resistant to inorganic isotopic exchange than carbonate oxygen, but carbonate oxygen is more resistant to microbially-mediated exchange [50].

**Figure 2:**
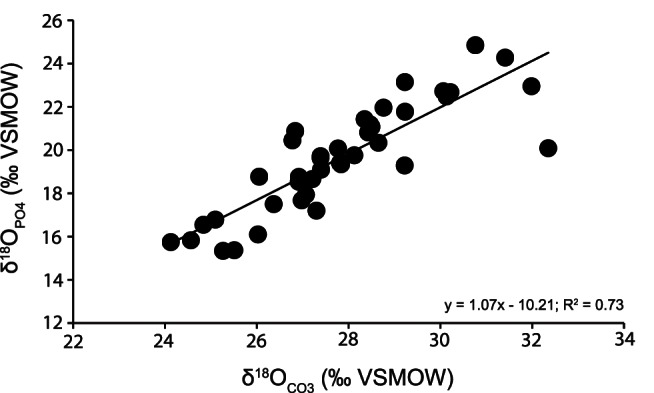
Regression line for mean δ^18^O_CO3_ and δ^18^O_PO4_ (‰ VSMOW) values. Each point represents mean isotopic value for each taxon per locality.

Modern, unaltered bioapatites exhibit a linear relationship between δ^18^O_CO3_ and δ^18^O_PO4_ with a consistent difference (δ^18^O_CO3_ - δ^18^O_PO4_ = ?^18^O_CO3-PO4_) of 8.6–9.1‰ for co-occurring CO_3_^−2^ and PO_4_^−3^ formed in isotopic equilibrium with body water at a constant temperature [51]–[53]. In this study, the mean ?^18^O_CO3-PO4_ was 8.2±1.3‰ (VSMOW), close to the expected value. Figure 2 shows the δ^18^O_PO4_-δ^18^O_CO3_ regression from this study. Zazzo *et al.* [50] suggested that the slope of the regression line between δ^18^O_CO3_ and δ^18^O_PO4_ is close to 1 in modern (unaltered) bioapatite. Slopes higher than unity suggest more extensive alteration of δ^18^O_CO3_ by inorganic mechanisms, whereas slopes lower than unity indicate a higher degree of microbially-mediated isotopic exchange of phosphate. Our slope is close to unity, but slightly higher (1.07). This slope is not as high as those observed by Zazzo *et al.* [50] in samples affected by intense diagenesis (see their Fig. 4) and no significant differences were detected by an ANCOVA test between our δ^18^O_PO4_-δ^18^O_CO3_ regression line and those proposed by Bryant *et al.* [52] and Iacumin *et al.* [53] (F = 0.473, p = 0.874).

These results suggest that our samples have experienced minimal isotopic alteration of either phosphate or carbonate oxygen. There are no comparable tests for carbon isotopes, but the fact that species cluster in bivariate isotope space, and that the relative positions of these clusters are consistent for some taxa, suggest that animal paleobiology, and not diagenesis, is the main driver of isotopic variation.

### Paleoecology of the Iberian Fossil Mammalian Taxa

**Figure 3:**
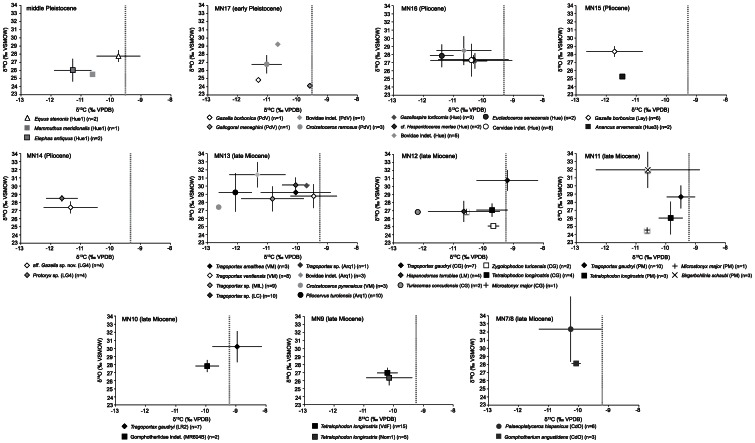
δ^18^O_CO3_ (‰ VSMOW) *versus* δ^13^C (‰ VPDB) for mammalian taxa in each MN and middle Pleistocene. Mean and standard deviation values are provided. Dashed grey line indicates the cut-off δ^13^C value between woodland-mesic C_3_ grassland and open woodland-xeric C_3_ grassland. CdO = Cerro del Otero, Nom1 = Nombrevilla 1, VdF = Los Valles de Fuentidueña, LR2 = La Roma 2, MR604B = Masía de la Roma 604B, PM = Puente Minero, LM = Los Mansuetos, CG = Cerro de la Garita, Arq1 = El Arquillo 1, LC = Las Casiones, MIL = Milagros, VM = Venta del Moro, LG4 = La Gloria 4, Lay = Layna, Hue3 = Huéscar 3, Hue = Huélago, PdV = La Puebla de Valverde, Hue1 = Huéscar 1. n is the number of sampled teeth.

In terrestrial settings, the dominant control on the δ^13^C value of plants is photosynthetic pathway [54]–[58]. Plants following the C_3_ or Calvin-Benson photosynthetic pathway (trees, shrubs, forbs and cool-season grasses) strongly discriminate against ^13^C during fixation of CO_2_, yielding tissues with δ^13^C values averaging −27‰ (VPDB) (ranging from −36 and −22‰). The most negative δ^13^C values of this range (−36 to −30‰) reflect closed-canopy conditions due to recycling of ^13^C-depleted CO_2_ and low irradiance. The highest values (−25 to −22‰) correspond to C_3_ plants from high light, arid, or water stressed environments. C_4_ plants (Hatch-Slack photosynthetic pathway) comprise grasses and sedges from areas with a warm growing season and some arid-adapted dicots. C_4_ plants discriminate less against ^13^C during carbon fixation, yielding mean δ^13^C value of −13‰ (ranging from −17‰ to −9‰). Crassulacean acid metabolism (CAM) is the least common pathway, occurring chiefly in succulent plants. CAM plants exhibit δ^13^C values that range between the values for C_3_ and C_4_ plants. Using the expected δ^13^C ranges for C_3_ and C_4_ plants and a typical diet-to-enamel fractionation of +14.1±0.5‰ [39], we can estimate the expected δ^13^C values for pure C_3_ feeders in different habitats (closed-canopy, −22 to −16‰; woodland-mesic C_3_ grassland, −16 to −11‰; open woodland-xeric C_3_ grassland, −11 to −8‰) and pure C_4_ feeders (−3‰ to +5‰). Enamel δ^13^C values between −8‰ and −3‰ represent mixed C_3_–C_4_ diets. When considering fossil taxa, however, it is necessary to account for shifts in the δ^13^C value of atmospheric CO_2_ (the source of plant carbon), including anthropogenic modification due to fossil fuel burning, which has decreased the δ^13^C value of atmospheric CO_2_ from −6.5 to −8‰ since onset of the Industrial Revolution [59]–[60]. Using isotopic data from marine foraminifera, Tipple *et al.* [40] reconstructed the δ^13^C value of the atmospheric CO_2_ since the Cretaceous. In order to calculate vegetation δ^13^C end-members, we considered the following time bins: late Miocene, Pliocene and Pleistocene. Table 2 shows a summary with δ^13^C_atmCO2_ and δ^13^C cut-off values for the transition between diets composed of different types of vegetation for the late Miocene, the Pliocene and the Pleistocene. The absolute cut-off δ^13^C value between woodland-mesic C_3_ grassland and open woodland-xeric C_3_ grassland is difficult to determine, but our threshold values are in agreement with previous studies. In this sense, Kohn *et al.* [61] suggested a threshold value of −9‰ between woodland and more open conditions when investigating a North American Pleistocene fossil site. Our C_3_ range also agrees well with Feranec *et al.* [62] who proposed a range of pure C_3_ δ^13^C values between −19.5‰ and −6.5‰, in a study focused on a Spanish Pleistocene fossil site. Matson *et al.* [63] compiled plant δ^13^C values from different types of modern ecosystems and our cut-off δ^13^C values for open woodland-xeric C_3_ grassland fit well with δ^13^C values for C_3_ trees, shrubs and grasses found mainly in Mediterranean forest, woodland and scrub, tropical and subtropical dry broadleaf forest, and desert and xeric shrubland, therefore pointing to some degree of aridity for that range of δ^13^C values. Figure 3 presents biplot δ^18^O_CO3_- δ^13^C graphs for each MN. Table 3 shows mean isotopic values for each taxon and their inferred dietary behaviour according to previous studies based on tooth morphology, microwear and isotopes. The whole isotopic dataset and statistical analyses are shown in Tables S1 and S4, respectively.

**Table 2:** δ^13^C of atmospheric CO_2_ (δ^13^C_atmCO2_) and mammalian enamel δ^13^C (δ^13^C_enamel_) cut-off values between different environments in the late Miocene, Pliocene and Pleistocene.

	Late Miocene	Pliocene	Pleistocene
δ^13^C_atmCO2_	−6.2	−6.3	−6.5
δ^13^C_enamel_ closed canopy forest	< −14.2	< −14.3	< −14.5
δ^13^C_enamel_ woodland to woodland-mesic C_3_ grassland	−14.2 to −9.2	−14.3 to −9.3	−14.5 to −9.5
δ^13^C_enamel_ open woodland-xeric C_3_ grassland	−9.2 to −6.2	−9.3 to −6.3	−9.5 to −6.5
δ^13^C_enamel_ mixed C_3_–C_4_ grassland	−6.2 to −1.2	−6.3 to −1.3	−6.5 to −1.5
δ^13^C_enamel_ C_4_ grassland	> −1.2	> −1.3	> −1.5

δ^13^C_atmCO2_ values are from Tipple *et al.* [40], δ^13^C_enamel_ have been calculated using a diet-to-enamel fractionation of 14.1‰ from Cerling & Harris [39]. All values are in ‰ VPDB.

**Table 3:** Site, MN, age (Ma), family, taxa, mean ±1 SD δ^13^C (‰ VPDB), δ^18^O_CO3_ (‰ VSMOW) and δ^18^O_ PO4_ (‰ VSMOW) values, inferred diet and references to other studies.

Site	MN	Age (Ma)	Family	Taxa	δ^13^C_CO3_ (‰ VPDB)	δ^18^O_CO3_ (‰ VSMOW)	δ^18^O_PO4_ (‰ VSMOW)	Diet	References
Huéscar 1	MP	0.80	Elephantidae	*Mammuthus meridionalis*	−10.6	25.5	15.4	Mixed feeder-Grazer	[64], [78]–[80]
Huéscar 1	MP	0.80	Elephantidae	*Elephas antiquus*	−11.3±0.6	26.0±1.4	16.1±1.4	Browser-Mixed feeder	[76], [77]
Huéscar 1	MP	0.80	Equidae	*Equus stenonis*	−9.8±0.7	27.8±0.7	20.1±0.1	Grazer	[70]
La Puebla de Valverde	MN17	2.13	Bovidae	*Gazella borbonica*	−11.3	24.8	16.5	Browser-Mixed feeder	[67]–[68], [70], [73]–[74]
La Puebla de Valverde	MN17	2.13	Bovidae	*Gallogoral meneghini*	−9.6	24.1	15.7	Mixed feeder	[82]
La Puebla de Valverde	MN17	2.13	Bovidae	Undetermined Bovidae	−10.7	29.2	21.8		
La Puebla de Valverde	MN17	2.13	Cervidae	*Croizetoceros ramosus*	−11.0±0.5	26.8±1.1	20.5	Browser	[64], [70]
Huélago	MN16	2.60	Bovidae	*Gazellospira torticornis*	−10.3±0.4	27.2±1.0	18.7±1.4	Mixed feeder	[70]
Huélago	MN16	2.60	Bovidae	cf. *Hesperidoceras merlae*	−10.3±1.2	27.4±0.5	19.7±0.5	Mixed feeder	J. Morales, pers. comm.
Huélago	MN16	2.60	Bovidae	Undetermined Bovidae	−10.7±0.9	28.5±1.8	21.2±3.0		
Huélago	MN16	2.60	Cervidae	Undetermined Cervidae	−10.4±1.4	27.3±2.0	17.2±2.1		
Huélago	MN16	2.60	Cervidae	*Eucladoceros senezensis*	−11.4±0.4	27.8±1.4	19.4±2.4	Oportunistic feeder	[75]
Huéscar 3	MN15	3.70	Gomphoteriidae	*Anancus arvernensis*	−11.5±0.02	25.3±0.1	15.3±0.3	Browser	[64], [72]
Layna	MN15	3.91	Bovidae	*Gazella borbonica*	−11.7±0.9	28.4±0.7	21.4±1.8	Browser-Mixed feeder	[67]–[68], [70], [73]–[74]
La Gloria 4	MN14	4.19	Bovidae	aff. *Gazella* sp. nov.	−11.3±0.9	27.4±0.7	19.6±2.5	Browser-Mixed feeder	[67]–[68], [70], [73]–[74]
La Gloria 4	MN14	4.19	Bovidae	*Protoryx* sp.	−11.6±0.5	28.5±0.4	21.1±0.7	Browser-Mixed feeder	[67], [74]
Venta del Moro	MN13	5.69	Bovidae	*Tragoportax amalthea*	−10.1±1.2	29.2±0.3	23.2±0.9	Mixed feeder with strong grazing habits	[66], [67]
Venta del Moro	MN13	5.69	Bovidae	*Tragoportax ventiensis*	−9.5±0.8	28.8±1.5	22.0±1.8	Mixed feeder with strong grazing habits	[66], [67]
Venta del Moro	MN13	5.69	Cervidae	*Croizetoceros pyrenaicus*	−12.6	27.4	19.1±0.8	Browser	[64], [70]
Milagros	MN13	5.69	Bovidae	*Tragoportax* sp.	−10.8±1.1	28.4±1.5	20.8±2.1	Mixed feeder with strong grazing habits	[66], [67]
Las Casiones	MN13	6.08	Bovidae	*Tragoportax* sp.	−10.1±0.5	30.1±0.9	22.5±0.9	Mixed feeder with strong grazing habits	[66], [67]
El Arquillo 1	MN13	6.32	Bovidae	*Tragoportax* sp.	−9.7	30.1	22.7	Mixed feeder with strong grazing habits	[66], [67]
El Arquillo 1	MN13	6.32	Bovidae	Undetermined Bovidae	−11.3±0.9	31.4±1.6	24.3±1.5		
El Arquillo 1	MN13	6.32	Cervidae	*Pliocervus turolensis*	−12.1±0.6	29.2±2.4	19.3±1.2	Browser	[64]
Cerro de la Garita	MN12	7.01	Bovidae	*Tragoportax gaudryi*	−9.2±1.0	30.8±1.3	24.9±0.6	Mixed feeder with strong grazing habits	[66], [67]
Cerro de la Garita	MN12	7.01	Mammutidae	*Zygolophodon turicensis*	−9.7±0.2	25.1±0.1	16.8±0.7	Browser-Mixed feeder	[64]
Cerro de la Garita	MN12	7.01	Gomphoteriidae	*Tetralophodon longirostris*	−9.7±0.5	27.1±0.8	17.9±0.4	Browser	[64]
Cerro de la Garita	MN12	7.01	Suidae	*Microstonyx major*	−10.6	26.9	18.8	Omnivore	[64]
Cerro de la Garita	MN12	7.01	Cervidae	*Turiacemas concudensis*	−12.2±0.1	26.8±0.2	20.9±0.9	Browser	J. Morales, pers. comm.
Los Mansuetos	MN12	7.01	Bovidae	*Hispanodorcas torrubiae*	−10.7±1.2	26.9±1.3	18.5±1.3	Browser-Mixed feeder	[68]
Puente Minero	MN11	7.83	Bovidae	*Tragoportax gaudryi*	−9.5±0.5	28.7±1.4	20.3±2.2	Mixed feeder with strong grazing habits	[66], [67]
Puente Minero	MN11	7.83	Gomphoteriidae	*Tetralophodon longirostris*	−9.8±0.4	26.1±2.0	18.8±1.3	Browser	[64]
Puente Minero	MN11	7.83	Suidae	*Microstonyx major*	−10.6	24.6	15.8	Omnivore	[64]
Puente Minero	MN11	7.83	Giraffidae	*Birgerbohlinia schaubi*	−10.6±1.7	32.0±2.2	23.0±1.8	Browser	J. Morales, pers. comm.
Masía de la Roma 604B	MN10	8.26	Gomphoteriidae	Undetermined Gomphotheriidae	−10.0±0.4	27.8±0.7	19.4±0.0	Browser	
La Roma 2	MN10	8.79	Bovidae	*Tragoportax gaudryi*	−9.0±0.8	30.2±1.9	22.7±2.4	Mixed feeder with strong grazing habits	[66], [67]
Los Valles de Fuentidueña	MN9	9.55	Gomphoteriidae	*Tetralophodon longirostris*	−10.2±0.3	27.0±0.6	17.7±0.7	Browser	[64]
Nombrevilla 1	MN9	10.87	Gomphoteriidae	*Tetralophodon longirostris*	−10.2±0.8	26.4±0.9	17.5±1.5	Browser	[64]
Cerro del Otero	MN7/8	11.13	Gomphoteriidae	*Gomphotherium angustidens*	−10.1±0.2	28.1±0.3	19.8±0.3	Browser	[64]
Cerro del Otero	MN7/8	11.13	Cervidae	*Palaeoplatyceros hispanicus*	−10.3±1.1	32.4±4.0	20.1±2.2	Browser	[64]

MP is middle Pleistocene. Age from Domingo *et al.* ([16], unpublished data).

### Late Miocene (Cerro del Otero, MN7/8–Venta del Moro, MN13)

Among Miocene proboscideans, *Gomphotherium angustidens* had brachyo-bunodont dentition, suggesting a browsing behaviour, which is in agreement with δ^13^C values pointing to consumption of woodland or woodland/C_3_ grassland vegetation. The gomphothere *Tetralophodon longirostris* replaced *Gomphotherium angustidens*. *Tetralophodon* was larger and more hypsodont than *Gomphotherium*, but also probably a browser [64]. Its δ^13^C values shift from lower values similar to *Gomphotherium* in older localities (Nombrevilla and Los Valles de Fuentidueña, MN9) to ∼0.5‰ higher values in younger sites (Puente Minero, MN11 and Cerro de la Garita, MN12). The mammutid *Zygolophodon turicensis* from the Cerro de la Garita locality had a zygodont dentition with sharp, transverse ridges and δ^13^C values similar to those for the youngest *Tetralophodon*. Overall, the slight trend of increasing δ^13^C values toward the end of the Miocene in these proboscideans points to consumption of plants from increasingly open, drier habitats. Since proboscideans are obligate drinkers [34], [65], the difference in δ^18^O_CO3_ and δ^18^O_PO4_ values likely reflects a change in the isotopic composition of ingested δ^18^O_w_ spatially or temporally. In this case, *Z. turicensis* has the lowest isotopic values, with intermediate values for *T. longirostris* and the highest values for *G. angustidens*. This might be indicating differences in the source of ingested water with *G. angustidens* drinking in more open settings (Fig. 3, Table 3).

**Figure 4:**
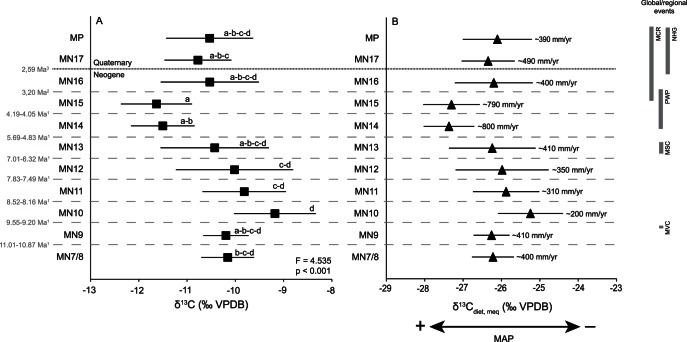
δ^13^C and δ^13^C_diet, meq_ (‰ VPDB) values across time bins. **A)** Mean and standard deviation δ^13^C (‰ VPDB) values in each MN. Letters indicate Tukeýs homogeneous groups. B) Mean and standard deviation δ^13^C_diet, meq_ (‰ VPDB) in each MN with mean annual precipitation (after Kohn [38]). Chronology according to ^1^Domingo *et al*. ([16], unpublished data), ^2^Agustí *et al.* [89], ^3^the onset of the Quaternary according to the chronology confirmed in 2009 by the International Union of Geological Sciences. The ages of the global/regional events are not absolute, but approximate according to the MN chronology. MCR = Mediterranean Climate Regime, NHG = Northern Hemisphere glaciation, PWP = Pliocene Warm Period, MSC = Messinian Salinity Crisis, MVC = Middle Vallesian Crisis.

In the case of Miocene bovids, the boselaphine *Tragoportax* is the best-represented genus. It had relatively long limbs suggesting cursorial adaptations and preference for open habitats [64]. Microwear studies performed on the teeth of this bovid suggest it was a mixed feeder with strong grazing habits [66]–[67]. This is consistent with its δ^13^C values, which are the highest for any taxon in all the MNs in which *Tragoportax* occurs (Fig. 3), and in most MNs are close to values expected for animals foraging in open woodlands or dry C_3_ grasslands. In the MN13 fossil sites, *Tragoportax* δ^13^C values were ∼1–2‰ lower, most likely due to a shift towards more humid conditions (see next section and Fig. 4). Using dental microwear, Merceron *et al.* [68] showed that a species of the bovid *Hispanodorcas* from the Neogene of northern Greece (*H. orientalis*) had strong similarities to extant browsers and mixed feeders; that reconstruction is also consistent with the δ^13^C values of *H. torrubiae* from Los Mansuetos (MN12; Fig. 3). According to Merceron *et al.* [69], *Tragoportax* was likely an obligate drinker based on a low inter-individual δ^18^O variability among species, and therefore its high δ^18^O_CO3_ and δ^18^O_PO4_ values when compared to the rest of taxa (including the bovid *H. torrubiae*) in MN10–12 (Fig. 3, Table 3) are consistent with ingestion of evaporated water in open environments.

Cervids have the lowest δ^13^C values of the late Miocene mammalian assemblage (Fig. 3), consistent with membership in the browsing guild as indicated by tooth morphology and microwear analyses [64], [70] (Table 3). The very low values for the cervids in MN12 and MN13 (between −12 and −13‰) point to foraging in a denser woodland, but not a closed canopy forest. Cervid δ^18^O_CO3_ and δ^18^O_PO4_ values yield different results with intermediate δ^18^O_CO3_ values (relative to other mammals), but consistently low δ^18^O_PO4_ values (Table 3). Cervids likely drank in the closed environments in which they foraged (which would yield low δ^18^O values). Therefore, the intermediate δ^18^O_CO3_ values point to some degree of alteration.

Like modern giraffes, although with a shorter neck, the giraffid *Birgerbohlinia schaubi* was likely a browser; this interpretation is supported by δ^13^C values indicative of woodland foraging. The very high δ^18^O_CO3_ and δ^18^O_PO4_ values in *B. schaubi* relative to other mammals from the Puente Minero (MN11) locality (and most other late Miocene mammals) (Fig. 3, Table 3) may indicate that this sivatherine obtained much of its water from highly evaporated leave water as suggested by Cerling *et al.* [71] for the extinct *Palaeotragus* and Levin *et al.* [65] for modern giraffids.

Finally, the suid *Microstonyx major* has intermediate δ^13^C values in the Puente Minero (MN11) and Cerro de la Garita (MN12) fossil sites. Suids are more omnivorous and according to Agustí and Antón [64], *M. major* had a cranial morphology suggesting a strong and highly mobile muzzle disk (like in modern pigs) interpreted as an adaptation to digging roots and tubers, although other sources of dietary intake such as fruits, insects and even carrion cannot be discarded, the combination of which may have given rise to the observed intermediate δ^13^C values.

### Pliocene (La Gloria 4, MN14–Huélago, MN16)

The gomphothere *Anancus arvernensis* has δ^13^C values indicative of browsing in a woodland to woodland-mesic C_3_ grassland (Fig. 3), which is consistent with observations by Agustí and Antón [64] and Tassy [72] who argued that its dentition was similar to that of other tetralophodont gomphotheres. Low δ^18^O_CO3_ and δ^18^O_PO4_ values may relate to ingestion of water in closed areas or flowing water not subject to significant evaporation (Fig. 3, Table 3).

The Pliocene bovids *Gazella* and *Protoryx* were ubiquitous taxa as far as occupancy of different habitats is concerned and are considered browsers to mixed feeders [67]–[68], [70], [73]–[74]; the relatively low δ^13^C values for these taxa are more supportive of a browsing habitat (Fig. 3, Table 3). Rivals and Athanassiou [70] argued that the antelope *Gazellospira torticornis* was a mixed feeder that grazed on seasonal or regional basis. Although this antelope has ∼1 to 1.5‰ higher δ^13^C values than *Gazella* and *Protoryx*, these values are consistent with woodland browsing and do not point to a substantial proportion of grass in the diet. The bovid cf. *Hesperidoceras merlae* has similar δ^13^C values to *G. torticornis* (Fig. 3, Table 3), supporting also woodland browsing. Pliocene bovid δ^18^O_CO3_ and δ^18^O_PO4_ values show a slight decrease towards younger sites related to a change in global conditions in the Pliocene (Table 3), but δ^18^O values agree well with the ingestion of non-evaporated waters.

The cervid *Eucladoceros senezensis* has the lowest δ^13^C value of the mammalian assemblage from the Huélago locality (MN16), although that value is still typical of a woodland and not of a closed canopy forest. *Eucladoceros* was a large-sized deer and, according to Croitor [75], it had an oportunistic feeding behaviour that allowed it to occupy more open environments as well as the more closed habitats typically used by cervids. Pliocene cervids from Huélago have similar δ^18^O_CO3_ and δ^18^O_PO4_ values to bovids, indicating a similar source of ingested water.

### Pleistocene (La Puebla de Valverde, MN17–Huéscar 1)

Filippi *et al.* [76] and Palombo *et al.* [77] studied microwear on *Elephas antiquus* of the Middle Pleistocene and suggested a browsing to mixed feeding behaviour; our δ^13^C data are consistent with woodland browsing but do not point to a substantial proportion of grass in the diet (Fig. 3). *Mammuthus meridionalis* has been considered to be a mixed feeder to grazer based on microwear and previous stable isotope analyses [78]–[80]. Our *M. meridionalis* δ^13^C value is more indicative of a mixed feeder occupying a woodland (Fig. 3).

The bovid, *Gallogoral meneghini* from La Puebla de Valverde (MN17) has higher δ^13^C values, close to those expected for an animal foraging in an open woodland (Fig. 3, Table 3). According to Guérin [81], Agustí and Antón [64] and Brugal and Croitor [82], *G. meneghini* was a mixed feeder with a robust skeleton and short limbs adapted to locomotion on mountainous uneven areas similar to modern gorals from Asia. Fakhar-i-Abbas *et al.* [83] studied the feeding preferences of the gray goral and found out that it relies mainly on grasses, although it can browse too; this is in agreement with our *G. meneghini* δ^13^C values situated towards the high cut-off for open woodland and mesic C_3_ grassland. Lower δ^13^C values in the case of *Gazella borbonica* are similar to those for this bovid in the Pliocene and again these values are consistent with woodland browsing and do not point to a substantial proportion of grass in the diet.

The cervid *Croizetoceros ramosus* also shows low δ^13^C values indicative of a woodland. The equid *Equus stenonis* has higher δ^13^C values near those expected for animals feeding in an open woodland (Fig. 3). This might be indicating ingestion of C_3_ grasses not subject to water stress. Slightly higher δ^18^O_CO3_ and δ^18^O_PO4_ values for the equid *E. stenonis* and the cervid *C. ramosus* in comparison to the elephantids and bovids may suggest ingestion of water in more open areas (in the case of the equid) or consumption of more evaporated water in leaves (in the case of the cervid) (Fig. 3, Table 3).

### Changes in δ^13^C Values

Figure 4 shows δ^13^C and modern equivalent δ^13^C values (δ^13^C_diet, meq_), which can be related to MAP (see material and methods section and Table S2) between MN7/8 and the middle Pleistocene.

A prominent faunal turnover event, known as the Middle Vallesian Crisis (ca. 9.6 Ma) [84] occurred in Western Europe between MN9 and MN10. This event is recognized by the replacement of humid-adapted taxa with taxa more adapted to drier conditions, and is associated with the replacement of evergreen subtropical woodlands by a seasonally adapted deciduous woodland as observed by Agustí and Moyà-Solà [85] and Agustí *et al.* [84] in the Vallès-Penedès Basin (North Eastern Iberian Peninsula). This event coincides with the Mi7 positive shift in benthic foraminifera δ^18^O values interpreted to reflect global cooling [86]–[87]. In Figure 4A, δ^13^C values of herbivorous mammals in the Iberian Peninsula increase between MN9 (Nombrevilla 1 and Los Valles de Fuentidueña) and MN10 (La Roma 2 and Masía de la Roma 604B), which may be related to a change towards drier conditions. δ^13^C_diet, meq_ values mirror tooth enamel δ^13^C values, with an increase observed between these MNs (Fig. 4B). MAP values (estimated after Kohn, [38]) dropped from ∼410 mm/yr to ∼200 mm/yr between MN9 and MN10. Böhme *et al.*, [13]), who used the ecophysiological structure of herpetofaunas in the Calatayud-Daroca Basin of Spain to estimate changes in MAP over the Miocene, also recognized a decrease in precipitation at 9.7–9.6 Ma. However, the decrease in the study of Böhme *et al.* [13] is greater than 1000 mm/yr in comparison with the ∼200 mm/yr decrease estimated here. The explanation for this large difference is unclear, but we note that the Kohn [38] method has relatively large error.

During MN13, the Messinian Salinity Crisis (MSC) in the Mediterranean Basin resulted from a sharp decrease in the marine water circulation from the Atlantic and culminated in the formation of thick evaporite deposits [8]. The lack of significant differences in mammal tooth enamel δ^13^C values between MN12 and MN13 (t = −1.285, p = 0.204) suggests that the MSC did not cause substantial modifications to terrestrial ecosystems, although a post-hoc Tukeýs test places the MN13 in groups a, b, c, and d (*versus* groups c and d for MN12) pointing to more humid conditions. However, and since we cannot unequivocally determine the synchrony between the chronology assigned to the MN13 localities considered in this study and the MSC, we regard this conclusion as preliminary pending more accurate datings. Ongoing paleomagnetic analyses in the MN13 Venta del Moro fossil site may modify the current chronology, which places this locality as contemporaneous to the MSC (J. Morales, pers. comm. 2013). Fauquette *et al.* [88] carried out an analysis of 20 pollen sequences in the Mediterranean realm and found no differences when comparing data before, during and after the MSC.

Mean tooth enamel δ^13^C values decrease sharply from MN13 to MN14, and the mean value in MN15 is lower still (Fig. 4A). The statistically significant drop in δ^13^C values during MN14 and MN15 may be related to the Pliocene Warm Period which began at ∼5 Ma and brought about more humid conditions in Europe [1], [64]. Figure 4B also shows a drop in δ^13^C_diet, meq_, which corresponds to an increase in MAP values of ∼400 mm/yr between MN13 (∼ 410 mm/yr) and MN14 and MN15 (∼ 800 mm/yr). The decrease in δ^13^C values in MN14 and MN15 is not biased by the type of taxa sampled, since in La Gloria 4 and Layna ubiquitous taxa such as *Gazella* and *Protoryx* were chosen and therefore, an isotopic change in these generalistic bovids [67]–[68], [70], [73]–[74] points towards real paleoenvironmental variations.

After MN15, δ^13^C values increase in MN16, MN17 and middle Pleistocene, but do not reach values as high as those observed in MN10, MN11 and MN12 (Fig. 4A). This increase in δ^13^C values corresponds to global and regional climatic changes and to faunal and environmental changes in Europe. The beginning of MN16 (∼3.2 Ma) [89] predates the onset of Northern Hemisphere glaciation [1], [90]. At that time, the modern Mediterranean climatic regime was established and aridity in Europe was enhanced, which led to changes in mammalian fossil assemblages in such a way that, according to Agustí *et al.* [89], the Villanyian mammal turnover occurred at this time with an increase in grazers, the appearance of morphological features associated with a highly cursorial lifestyle in some ungulates, and the diversification of pursuit carnivores. All of these changes point towards the development of prairies and grasslands in Europe [64], [89]. Fortelius *et al.* [91] estimated hypsodonty index in mammalian herbivores between the Late Miocene and the Pliocene in Eurasia and found out that browsing taxa in MN15 were replaced by grazers in MN16 and MN17. Another important event occurred at ∼2.6 Ma, when there was a replacement of forests by tundra-like vegetation in northern and central Europe, while in northwestern Africa, savanna biome shrunk in favour of desert biome [64]. The Iberian Peninsula also experienced a shift towards the development of more herbaceous vegetation, such as the well-documented increase of *Artemisia* [11], [92]. The increase in mammal tooth enamel δ^13^C values observed in MN16, MN17 and the middle Pleistocene may reflect this episode.

### Temperature Record

**Figure 5:**
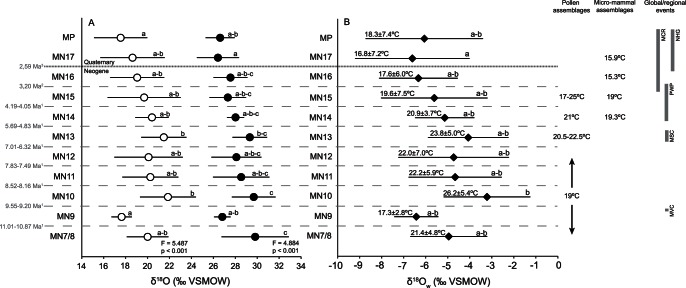
δ^18^O_CO3_ and δ^18^O_PO4_ (‰ VSMOW) values across time bins. **A)** Mean and standard deviation δ^18^O_CO3_ (black circles) and δ^18^O_PO4_ (white circles) (‰ VSMOW) values. Letters indicate Tukeýs homogeneous groups. B) Mean and standard deviation δ^18^O_w_ (‰ VSMOW) and MAT (°C) values calculated by applying the equation (3) of Rozanski *et al.* [41]. MAT values based on pollen and micro-mammal data are from Fauquette *et al.* [88], [95], Hernández Fernández *et al.* [10] and Jiménez-Moreno *et al.* [11]. Chronology according to ^1^Domingo *et al.* ([16], unpublished data), ^2^Agustí *et al.* [89], ^3^the onset of the Quaternary according to the chronology confirmed in 2009 by the International Union of Geological Sciences. The ages of the global/regional events are not absolute, but approximate according to the MN chronology. MCR = Mediterranean Climate Regime, NHG = Northern Hemisphere glaciation, PWP = Pliocene Warm Period, MSC = Messinian Salinity Crisis, MVC = Middle Vallesian Crisis.

Figure 5 shows the variations in tooth enamel δ^18^O_CO3_ and δ^18^O_PO4_ values (Fig. 5A), and δ^18^O_w_ values and mean annual temperature (MAT) (Fig. 5B) estimated using the taxon-specific relationships (Table S3) and equation (3) from Rozanski *et al.* [41]. The Mi7 cooling event associated with the Middle Vallesian Crisis (between MN9 and MN10) is not evident in the tooth enamel δ^18^O values. Instead, δ^18^O values increase between MN9 and MN10, suggesting an increase in MAT (Fig. 5B). Based on pollen assemblages from the Iberian Peninsula, Jiménez-Moreno *et al.* [11] estimated that MAT during the Tortonian (MN7/8 to the middle of MN12) was 19°C. The mean MAT estimate from MN7/8 to MN12 in our study is slightly warmer, 21.8±3.2°C. Van Dam & Reichart [93] analyzed δ^18^O_CO3_ values on equid tooth enamel to estimate δ^18^O_w_ and MAT. They obtained a mean MAT of 15.4±2.1°C between MN9 and MN12, substantially lower than the values estimated here.

Jiménez-Moreno *et al.* [11] argued that during the Messinian, there were not major variations in climate before, during and after the MSC. The pollen assemblage from the Carmona section suggests a MAT between 20.5°C and 22.5°C during the Messinian in southwestern Spain. In our study, MN13 fossil sites that correspond to the Messinian suggest a warmer MAT of 23.8±5.0°C (Fig. 5B). Matson & Fox [94] estimated MAT using equid tooth enamel δ^18^O_PO4_ values and found an increase from 15.5°C for MN12 sites (Los Mansuetos and Concud) to 21.4°C for MN13 sites (Venta del Moro, Librilla, Molina de Segura and La Alberca). Van Dam & Reichart [93] obtained MAT values of 12.9°C for MN13, again much lower than other studies.

Fauquette *et al.* [88], [95] estimated MAT using pollen assemblages in the Mediterranean realm from the early Pliocene (∼MN14). Assemblages from the Andalucía G1 section indicate a MAT of 21°C. Tooth enamel δ^18^O values from MN14 localities in our study yield a comparable MAT of 20.9±3.7°C. Hernández Fernández *et al.* [10] used the bioclimatic analysis of Pliocene and Pleistocene rodent assemblages in the Iberian Peninsula and estimated a MAT of 19.3° during MN14, slightly lower than the estimates based on pollen assemblages and our data. The lowest MAT estimates for MN14 were from the isotopic studies by Matson & Fox [94] and van Dam & Reichart [93], who suggested MAT values of 16.1°C and 14.1°C, respectively.

Our estimate of MAT during MN15 is 19.6±7.5°C, in good agreement with that based on pollen from the Tarragona E2 section (17 to 25°C from 5.32 to 3 Ma) [11]. The estimates of Hernández Fernández *et al.* [10] based on rodent assemblages from MN15 (∼19°C) are also in good agreement.

After MN15, MAT values decrease, reflecting global cooling with the onset of the Northern Hemisphere glaciation at ∼2.7 Ma. Tooth enamel δ^18^O values from MN16 and MN17 in our study supplied MAT values of 17.6±6.0°C and 16.8±7.2°C respectively, slightly warmer than MAT values estimated by Hernández Fernández *et al.* [10] between MN16 (15.3°C) and MN17 (15.9°C). Once again, van Dam & Reichart [93] obtained the lowest MAT record for MN17 of 8.9°C. Nevertheless, the comparison of MAT values among studies that considered different fossil sites with ages younger than ∼2.7 Ma might be complicated by glacial-interglacial dynamics, which may have produced large shifts in temperature in relatively short periods of time.

Overall, the MAT values estimated here using mammalian tooth enamel are in good agreement with data from palynology and rodent assemblage analyses. Other isotopic studies on mammal tooth enamel from the Iberian Peninsula [93]–[94] showed consistently lower MAT values compared to those obtained here. This may be due to the use of different equations relating MAT and δ^18^O_w_. We use the equation (3) of Rozanski *et al.* [41], whereas Matson & Fox [94] and van Dam & Reichart [93] applied MAT-δ^18^O_w_ equations from meteorological stations near the location of the fossil sites. As previously highlighted, during the span of time considered in this study (late Miocene-middle Pleistocene), climate regimes shifted, and the modern Mediterranean regime was established at some point between ∼3.4 and 2.5 Ma. Hence, a worldwide meteorological MAT-δ^18^O_w_ equation integrating data from a range of climate regimes may constitute a better basis for estimating MAT than equations integrating a narrower range of climate regimes derived from local meteorological MAT-δ^18^O_w_ data. However, the differences in reconstructed MAT based on δ^18^O values of mammalian bioapatite for the same intervals highlight the sensitiviy of these reconstructions to both sampling and the assumptions behind the reconstructions.

### Absence of C_4_ Vegetation in Southwestern Europe

Our δ^13^C record offers no evidence of the high δ^13^C values typical of C_4_ consumers (Figs. 3 and 4, Table 2) and the calculation of the percentage of C_4_ vegetation points to a low C_4_ dietary intake (<20%) in most of the analyzed taxa. This percentage of C_4_ vegetation may reflect either an actual small fraction of C_4_ plants in mammal diets or it may be an artifact related to the ingestion of C_3_ plants from open areas subject to water stress (which therefore have higher δ^13^C values). The lack of a significant expansion of C_4_ plants in the Iberian Peninsula is intriguing. The expansion of C_4_ plants took place between 9 and 2 Ma in different regions [6]. C_4_ photosynthesis is favored under conditions of low atmospheric CO_2_, when growing seasons experience high temperature (i.e., summer rainfall), in arid regions, or in soils with high salinity. The combined effects of fires and herbivory may also lead to open environments where C_4_ grasses may thrive. Given the high temperatures suggested by our isotopic analyses (Fig. 5) and other proxy data, conditions in the late Miocene and early Pliocene would seem conducive to a regional C_4_ expansion if habitats were relatively open and there was adequate summer precipitation.

Palaeoclimatic studies of Iberian mammalian assemblages from late Miocene to middle Pleistocene (∼11.1 to 0.8 Ma) indicate that the most likely biomes at some of the fossil sites studied here (Puente Minero, Los Mansuetos, Cerro de La Garita, El Arquillo, Venta del Moro, La Gloria 4, Layna and Huéscar 1) were tropical deciduous woodland with perhaps occasional savanna and subtropical desert environments, prior to the development of the sclerophyllous woodland-shrubland at the start of the Pleistocene [10], [48]. By definition, a woodland supports woody cover of >40% and <80% with the remaining patches often dominated by grasses, either C_3_ or C_4_ [96]–[97]. In a study of the isotopic composition of individual pollen grains from ∼20 to 15 Ma in the Rubielos de Mora Basin, Urban *et al.* [98] showed that while the overall abundance of grass pollen was low and in the range expected for a woodland (10–15%), C_4_ grasses comprised 20–40% of the grains. Since there are no isotopic studies on pollen grains in the time interval selected for our study, we assume that C_4_ grasses were potentially present in the flora of the Iberian Peninsula since at least the Early Miocene.

While a detailed analysis of the ultimate cause/s for the low abundance of C_4_ plants in southwestern Europe after their expansion elsewhere is beyond the scope of this paper, there are several potential explanations. At middle latitudes, only regions with summer rainfall are suitable for C_4_ grasses. A seasonality of rainfall similar to the modern Mediterranean precipitation pattern, with precipitation occurring chiefly during the winter, would lead to very low abundance of C_4_ plants on the Iberian Peninsula. Several studies have questioned the age of 3.4 and 2.5 Ma for the onset of the Mediterranean climate and proposed that such a climate regime may have been present much earlier (e.g., [99]). For example, Axelrod [100] studied fossil leaves in the Mediterranean area and argued that sclerophyllous evergreen woodlands with chaparral undergrowth were present throughout the Miocene. Yet there is no way to determine if these species were dominant on the landscape, and Axelrod ([100]: p. 325) himself noted that sclerophyllous species might constitute part of the tropical-subtropical woodlands understory but that the “existence of chaparral and macchia over wide areas as climax vegetation in the Tertiary seems unlikely”.

Tzedakis [99] reviewed evidence for the onset of the Mediterranean climate regime and noted that seasonality similar to the summer-dry and winter-wet pattern may have appeared intermittently before the onset of the “true”-Mediterranean climate regime. The occasional occurrence of Mediteranean-like climate in the Iberian Peninsula in the early Pliocene has also been suggested by studies of rodent faunas and has been linked to the presence of bimodal precipitation regimes, which may produce a short summer dry season in addition to the winter dry season typical of tropical climates [10]. The prevalence of these short summer dry periods is probably not sufficient to explain the absence of C_4_-dominated landscapes.

An alternative is that C_4_ plants were somewhat more abundant, but that mammals selectively foraged on C_3_ plants, perhaps avoiding C_4_ plants because of their lower nutritional value [101]. Paleoecological studies from other regions suggest that this explanation is unlikely. In North America, South America, Asia and Africa (see a review in Strömberg [6]), when C_4_ plants became available (as determined by soil carbonates and other lines of evidence), they came to comprise a substantial part of the diet of at least some mammalian grazers. Indeed, once C_4_ grass became abundant, different taxa began to specialize on them. There is no reason to assume that some genera of Miocene mammals (e.g., *Tragoportax*, a mixed feeder with strong grazing habits) in the Mediterranean region would not have used a new dietary resource such as C_4_ grasses had they been abundant.

It seems that the most likely cause for a limited C_4_ vegetation development may be related to the biome configuration of the late Miocene-Pliocene in the Iberian region. Pollen records indicate low percentages (10–15%) of grasses, belonging to the Poaceae family, during the late Miocene and the Pliocene (Jiménez-Moreno, pers. comm. 2012). Pollen analyses are not able to distinguish between C_3_ and C_4_ grasses, but if we assume that the percentage of C_4_ plants estimated by Urban *et al.* [98] for the early Miocene Rubielos de Mora Basin (20–40%) was maintained in the late Miocene and Pliocene, the final percentage of C_4_ grasses may have not been enough as to be recorded on mammalian tooth enamel δ^13^C values.

### Conclusions

Long stratigraphic sequences of isotopic data from mammalian tooth enamel are not frequently analyzed due to gaps in the terrestrial fossil record. Such studies are important since they can reveal modifications in paleoenvironmental and paleoclimatic factors in terrestrial settings during critical intervals in Earth history. Here, we used stable isotope analysis of a succession of mammals from 18 localities in Spain ranging in age from 11.1 to 0.8 Ma to reconstruct environmental and climatic changes during the late Neogene and early Quaternary. In general, tooth enamel δ^13^C values indicate that analyzed taxa may have occupied woodland to mesic C_3_ grassland and in some cases, open woodland to xeric C_3_ grassland, with no evidence of significant C_4_ consumption in any of the genera we studied. An increase in δ^13^C values between MN9 and MN10 appears to correspond to the Middle Vallesian Crisis, a faunal turnover that led to the replacement of humid-adapted taxa by taxa more adapted to drier conditions. A significant decrease in δ^13^C values during MN14 and MN15 is probably linked to the Pliocene Warm Period (with an associated increase in moisture), whereas the higher δ^13^C values from MN16 onwards may have been a consequence of the increased aridity in Europe related to the onset of Northern Hemisphere glaciation. The MAT pattern estimated using tooth enamel δ^18^O_PO4_ values agrees well with the thermal trend based on palynological records, rodent assemblage structure, and other isotopic studies from the Iberian Peninsula, with a gradual drop in MAT from MN13 onwards in response to the progressive cooling observed since the Middle Miocene and culminating in the Northern Hemisphere glaciation.

## Acknowledgments

We are indebted to L. Alcalá and E. Espílez (Fundación Conjunto Paleontológico de Teruel-Dinópolis, Teruel) and P. Pérez (Museo Nacional de Ciencias Naturales-CSIC, Madrid) for kindly providing access to the studied material. S. D. Matson (University of Minnesota, now at Boise State University), and D. Andreasen, J. Lehman and J. Karr (University of California Santa Cruz) are acknowledged for help with isotopic analyses. We are grateful to G. Jiménez-Moreno (Universidad de Granada) for valuable information about Iberian pollen records, and J. Morales (Museo Nacional de Ciencias Naturales-CSIC) for clarification about the diet of some taxa and valuable comments that helped to improve the manuscript. We also thank the editor R.J. Butler for manuscript management.

## Supporting Information

Table S1 **Site, MN, age (Ma), signature, family, taxa, tooth, δ^13^C_CO3_ (‰ VPDB), δ^18^O_CO3_ (‰ VSMOW) and δ^18^O_PO4_ (‰ VSMOW) values for the whole set of fossil mammals from the Iberian Peninsula.** Age from Domingo et al. [16, unpublished data]. In the “Tooth” column: M = molar, P = premolar, superscript = upper teeth, subscript = lower teeth.
(XLS)

Table S2 **δ^13^C_enamel_ (‰ VPDB) values of the whole set of Iberian mammalian fossil tooth enamel.** ^1^δ^13^C_diet_ (‰ VPDB) calculated by using the offset of 14,1‰ between δ^13^C_enamel_ and δ^13^C_diet_ proposed by Cerling and Harris [39]. ^2^δ^13^C_atmCO2_ (‰ VPDB) is from Tipple et al. [40]. ^3^δ^13^C_diet, meq_ (‰ VPDB) was calculated using equation (2) (see text) and using the modern δ^13^C_atmCO2_ (‰ VPDB) of -8‰.
(XLS)

Table S3 **Equations used to calculate δ^18^O_w_ values from mammalian tooth enamel δ^18^O_PO4_ values.**
(XLS)

Table S4 **Statistical analyses comparing different mammalian taxa per MN.** Student-t test was used for those MNs where we sampled two genera, whilst ANOVA test was used for those MNs with more than 2 genera. Significant differences are highlighted in bold.
(XLS)
